# Fast Analytic Simulation for Multi-Laser Heating of Sheet Metal in GPU

**DOI:** 10.3390/ma11112078

**Published:** 2018-10-24

**Authors:** Daniel Mejia-Parra, Diego Montoya-Zapata, Ander Arbelaiz, Aitor Moreno, Jorge Posada, Oscar Ruiz-Salguero

**Affiliations:** 1 Laboratory of CAD CAM CAE, Universidad EAFIT, Cra 49 no 7-sur-50, 050022 Medellín, Colombia; dmejiap@eafit.edu.co (D.M.-P.); dmonto39@eafit.edu.co (D.M.-Z.); oruiz@eafit.edu.co (O.R.-S.); 2 Vicomtech, Paseo Mikeletegi 57, Parque Científico y Tecnológico de Gipuzkoa, 20009 Donostia/San Sebastián, Spain; aarbelaiz@vicomtech.org (A.A.); jposada@vicomtech.org (J.P.)

**Keywords:** multi-beam laser, heat transfer analysis, fast simulation, GPU, analytic solution

## Abstract

Interactive multi-beam laser machining simulation is crucial in the context of tool path planning and optimization of laser machining parameters. Current simulation approaches for heat transfer analysis (1) rely on numerical Finite Element methods (or any of its variants), non-suitable for interactive applications; and (2) require the multiple laser beams to be completely synchronized in trajectories, parameters and time frames. To overcome this limitation, this manuscript presents an algorithm for interactive simulation of the transient temperature field on the sheet metal. Contrary to standard numerical methods, our algorithm is based on an analytic solution in the frequency domain, allowing arbitrary time/space discretizations without loss of precision and non-monotonic retrieval of the temperature history. In addition, the method allows complete asynchronous laser beams with independent trajectories, parameters and time frames. Our implementation in a GPU device allows simulations at interactive rates even for a large amount of simultaneous laser beams. The presented method is already integrated into an interactive simulation environment for sheet cutting. Ongoing work addresses thermal stress coupling and laser ablation.

## 1. Introduction

Multi-beam laser heating of sheet metal is a relevant metalworking technique which has arisen interest of researchers in the last years. In contrast to single-beam heating, multi-beam heating provides two main advantages to the former: (1) the ability to process different locations of the sheet simultaneously [1], and (2) control of thermal stress levels by specific multi-beam configurations [2]. Industrial applications of multi-beam heating of sheet metal include laser forming and bending, laser cutting and additive manufacturing.

Thermal simulation is crucial for temperature and stress analysis of manufactured pieces. An adequate selection of laser parameters and a correct path planning allows for improving the efficiency of the process and minimizes material damage and waste. Current simulation approaches rely on numerical schemes which require fine geometry and time discretizations. Such discretizations imply high computational costs, which limit the application of these approaches in real manufacturing scenarios with large time/space domains and complex laser trajectories [3].

This manuscript presents a simulation approach for the multi-beam laser heating problem based on an analytic solution to the heat equation on rectangular domains. This analytic solution does not require a mesh nor a fine time discretization to solve the problem. As a consequence, our algorithm is able to run complex simulations with large time/space domains and complex multi-laser trajectories at interactive time rates. Furthermore, each laser beam trajectory is allowed to be independent from the others, with different time-dependent parameters, trajectories and time frames (i.e., each laser beam can be turned on/off independently at any point of the simulation).

The remainder of this manuscript is organized as follows: Section 2 discusses the relevant literature. Section 3 presents the mathematical model and describes the implementation of the proposed algorithm. Section 4 discusses the obtained results for different test cases. Finally, Section 5 presents the conclusions and introduces the future work.

## 2. Literature Review

### 2.1. Multi-Beam Single Trajectory vs. Multiple-Trajectory Simultaneous Laser Heating

There are currently two main applications for multi-laser heating in laser machining: (1) single-trajectory multi-laser heating and (2) multi-trajectory multi-laser heating.

In single-trajectory multi-beam laser heating, a leading laser is followed by a pattern of secondary finishing ones, all in the same trajectory (or with minimum spatial offset). Experimental evidence has shown that such a configuration reduces the thermal stresses produced by the laser beams in laser cutting (compared to single-beam cutting) [4]. Furthermore, specific configurations of the laser beams have been shown to reduce the required pressure of assisting gas [5]. Each laser beam may be produced by an independent source [6] or by diffraction of a single beam source [7].

On the other hand, in multi-trajectory heating, each laser beam follows an independent trajectory [1]. Multi-trajectory heating is relevant as it improves the machining times by processing different zones of the sheet at the same time. This manuscript focuses on the simulation of multi-trajectory laser heating.

### 2.2. Thermal Simulation for Sheet Metal Laser Heating

The problem of laser beam heating simulation has been widely researched for single-beam applications. Numerical methods are the most common simulation approach. Methods such as the Finite Differences Method (FDM) [8,9,10,11] and the Boundary Element Method (BEM) [12,13] have been used in the literature to study the thermal behaviour of sheet laser heating. However, these methods impose several numerical limitations such as dense rectangular grids in the case of FDM and non-sparse linear systems for BEM, requiring a high amount of computational resources even for simple problems.

The Finite Element Method (FEM) is a standard numerical approach for the simulation of physical problems. Nonlinear FEM has been applied to study the thermal/stress behaviour of the single-beam laser heating of rectangular sheets [14,15,16,17,18]. In contrast to FDM and BEM, FEM works by discretizing the domain using different types of meshes which allow fine discretizations (high level of detail) near interest zones (laser spot and trajectory) and coarse resolutions in other zones resulting in less expensive systems of equations. To address laser ablation and material removal, methods such as element birth and death [19], volume fractions [20], temperature thresholds [21,22,23], and the enthalpy method [15,16,17,18,24] are coupled to the different numerical schemes.

The Finite Volume Method (FVM) has been recently introduced in the literature for the study of laser ablation and sheet heating [25,26]. Contrary to previous numerical methods, the FVM allows to accurately model and simulate interactions between the laser beam and the sheet in its physical states: solid, liquid and gas. However, these interactions are highly nonlinear and, as a consequence, computationally expensive.

Numerical methods provide tools to simulate nonlinear interactions, phase changes, laser ablation and material removal. However, they are also computationally expensive, limiting the applications of the algorithms in real manufacturing scenarios with complex laser trajectories and large space/time domains. Analytical methods do not have such limitations, allowing fast simulation of complex problems at the cost of some simplifications of the physical model. Uni-dimensional analytic models have been implemented for the simulation of laser drilling processes for static laser beams [27,28]. A 3D model for laser heating of sheet metal for straight line trajectories is presented in [29], while 2D models for arbitrary laser trajectories have been presented in [30,31].

Despite the large amount of literature concerning single-beam laser heating, simulation for multi-beam laser processing has been rarely studied. In [6,32], the FDM is used to analyze the thermal and structural impact of two independent laser beams melting a sheet metal. On the other hand, in [2,33], a thermal/stress analysis of multi-beam laser heating is performed using FEM. These simulations show that multi-beam heating reduce the thermal stresses afflicted to the material. A semi-analytic model for the steady multi-beam laser heating problem is presented in [34]. This pseudo-analytic model is also implemented inside an optimization algorithm which estimates the best laser parameters for a given manufacturing process. The use of analytic solutions is crucial for optimization due to the optimization process being expensive per se, requiring multiple evaluations of the temperature fields with different laser parameters and/or trajectories.

### 2.3. Conclusions of the Literature Review

Contrary to single-beam, multi-beam heating is scarce in the literature. Numerical (as opposed to analytical) methods are computationally expensive. As a consequence, these methods are unable to simulate real world sheet sizes and laser trajectories.

This manuscript offers the implementation of an analytic Fourier-based method to simulate multi-trajectory laser heating. The characteristics of the implemented method are: (a) constant material properties, (b) natural convection, (c) simplification of the sheet into a 2D domain, (d) transient (time-history) temperature, (e) multiple laser head configurations, (f) independent laser trajectories (with independent parameters), and (g) arbitrary power on/off time history on each trajectory.

Our algorithm is heavily based on the previous work presented in [31]. However, our method presents several improvements to this previous work as follows: (a) our algorithm reformulates the analytic solution to allow simultaneously multiple laser beams instead of only one. (b) Our method allows completely asynchronous laser beams (independent parameters, trajectories and time frames) which is not a problem for single-beam approaches and has not been addressed in other multi-beam related work. (c) Instead of computing and storing the full temperature history, our implementation exploits the frequency-based solution approach by interactively providing the temperature only when requested by the user (even in non-monotonic order). (d) Computing the solution in the frequency domain allows arbitrary sheet discretizations for visualization and analysis, including triangular meshes, rectangular grids, polylines or sets of sampled points on the sheet. (e) Our GPU implementation of the algorithm using on-chip (local) memory presents an increased 10x speed-up on computation time over previous work.

## 3. Methodology

### 3.1. Heat Transfer Equation for Multi-Beam Laser Heating

The temperature distribution $u=u(\vec{x},t)$ of a 2D rectangular sheet satisfies the following partial differential equation:

(1) $$\begin{array}{c} \rho c_{p}\frac{\partial u}{\partial t}-\nabla \cdot \left(\kappa \nabla u\right)=\frac{F-q}{\Delta z},\\ q=h(u-u_{\infty }), \end{array}$$

where ρ, $c_{p}$ and κ are the material density, specific heat and thermal conductivity, respectively. $\nabla \cdot \nabla =\partial^{2}/\partial^{2}x+\partial^{2}/\partial^{2}y$ is the 2D Laplace operator. Δz is the thickness of the sheet. $F=F(\vec{x},t)$ is the set of surface heat sources affecting the sheet, and q=q(u) is the temperature-dependent convection on the sheet surface with its respective convection coefficient h≥0 and ambient temperature $u_{\infty }\in R$. For the previous partial differential equation, the following boundary and initial conditions are imposed on the sheet:

(2) $$\begin{array}{c} \begin{array}{c} {u|}_{x=0}{=u|}_{x=a}=u{{|}_{y=0}=u|}_{y=b}=u_{\infty },\\ u\left(\vec{x},0\right)=u_{\infty }. \end{array} \end{array}$$

**Figure 1:**
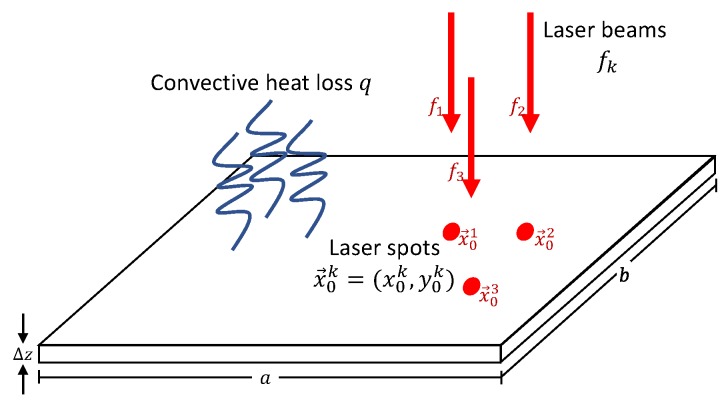
Multi-beam laser heating scheme. The sheet surface is heated by a set of laser beams $f_{1},f_{2},\cdots$ and cooled down due to natural convection *q*.

For the multi-beam approach, the set of heat sources *F* is defined as the sum of the heat inputs of each laser beam $f_{k}$:

(3) $$\begin{array}{c} F\left(\vec{x},t\right)=\sum_{k=1}^{num\_lasers}f_{k}\left(\vec{x},t\right),\\ f_{k}\left(\vec{x},t\right)=\left\{\begin{array}{cc} \frac{P_{k}\left(1-R\right)}{\pi r_{k}^{2}}, & ∥\vec{x}-{\vec{x}}_{0}^{k}{\left(t\right)∥}_{\infty }<\frac{r_{k}\sqrt{\pi }}{2},\\ 0, & \mathrm{otherwise}, \end{array}\right. \end{array}$$

where $P_{k}\geq 0$ is the power of the laser beam, $r_{k}>0$ is the radius of the laser spot and ${\vec{x}}_{0}^{k}\left(t\right)\in R^{2}$ is the center of the laser spot at time *t*. 0≤R<1 is the reflectivity of the material. $∥\vec{x}{∥}_{\infty }=\max\left(x,y\right)$ is the infinity norm in $R^{2}$. The above laser model transforms the circular laser spot with radius $r_{k}$ to its equivalent squared spot with area $\pi r_{k}^{2}$. We apply such transformation in order to develop the analytic solution in the next section. Figure 1 presents a scheme of the multi-beam laser heating problem.

### 3.2. Analytic Solution

Following the same procedure as in [31], the temperature *u* on the rectangular sheet can be expressed as a linear combination of Fourier functions:

(4) $$u\left(\vec{x},t\right)=u_{\infty }+\sum_{i=1}^{\infty }\sum_{j=1}^{\infty }\Theta_{ij}\left(t\right)X_{i}\left(x\right)X_{j}\left(y\right),$$

with basis:

(5) $$\begin{array}{c} X_{i}\left(x\right)=\sin\frac{i\pi x}{a},\\ Y_{j}\left(y\right)=\sin\frac{j\pi y}{b}. \end{array}$$

Applying separation of variables, the Fourier coefficients $\Theta_{ij}$ from Equation (4) can be expressed as a sum of the pseudo-coefficients $\theta_{ij}^{k}$ of each independent heat source:

(6) $$\Theta_{ij}\left(t\right)=\sum_{k=1}^{num\_lasers}\theta_{ij}^{k}\left(t\right),$$

where each laser beam $f_{k}$ defines its respective pseudo-coefficient $\theta_{ij}^{k}$ as follows:

(7) $$\theta_{ij}^{k}\left(t\right)=\theta_{ij}^{k}\left(t_{0}\right)e^{-\omega_{ij}\left(t-t_{0}\right)}+\frac{4}{ab\rho c_{p}\Delta z}\int_{t_{0}}^{t}\int_{0}^{b}\int_{0}^{a}f_{k}\left(\vec{x},\tau \right)X_{i}\left(x\right)Y_{j}\left(y\right)e^{-\omega_{ij}\left(t-\tau \right)}dxdyd\tau$$

with corresponding Laplacian eigenvalues:

(8) $$\omega_{ij}=\frac{\kappa }{\rho c_{p}}\left(\frac{i^{2}\pi^{2}}{a^{2}}+\frac{j^{2}\pi^{2}}{b^{2}}\right)+\frac{h}{\rho c_{p}\Delta z}.$$

Equation (7) is written recursively in terms of a previous known solution $\theta_{ij}^{k}\left(t_{0}\right)$. Therefore, each laser trajectory is discretized as a piecewise linear trajectory ${\vec{x}}_{0}^{k}=\left[{\vec{x}}_{0}^{k}\left(0\right),{\vec{x}}_{0}^{k}\left(t_{1}\right),{\vec{x}}_{0}^{k}\left(t_{2}\right),\cdots \right]$. In contrast to previous work [31], Equations (3), (6) and (7) have been reformulated to account for the multiple asynchronous laser beams. Finally, the closed form for the integral term in Equation (7) has been already presented in [31].

### 3.3. Algorithm

**Figure 2:**
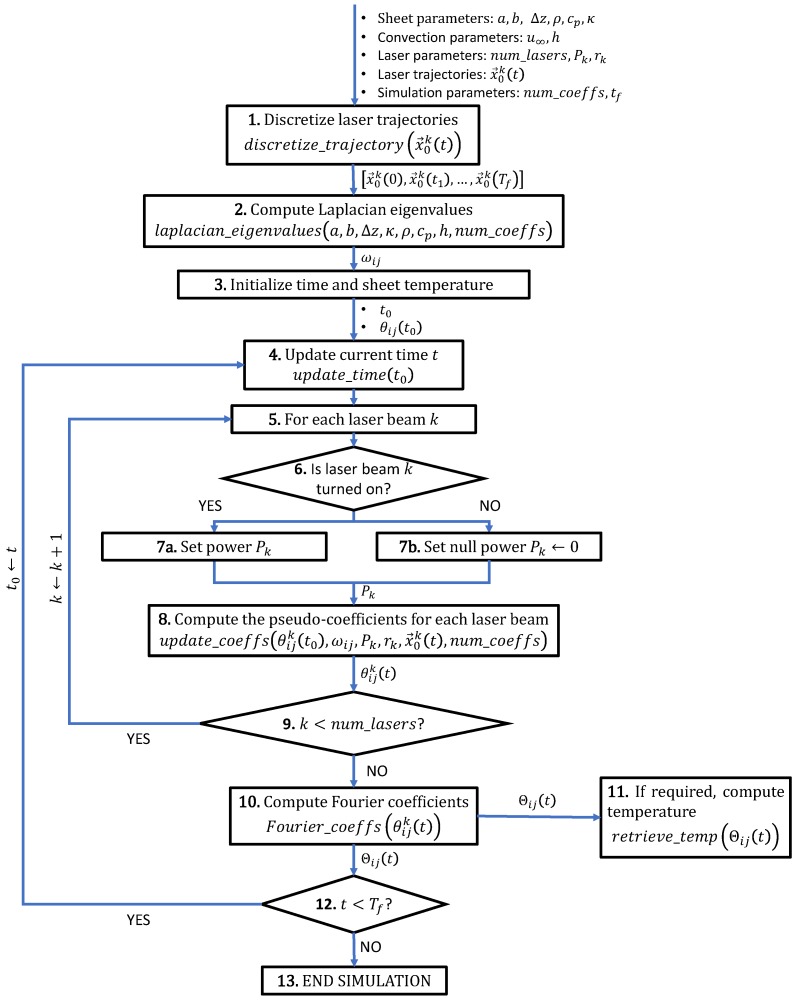
Diagram of the analytic multi-laser simulation algorithm.

Figure 2 presents a diagram of the simulation algorithm based on the analytic solution presented in Section 3.2. Each step of the algorithm is discussed in detail below:

1. **Discretize laser trajectories:** As discussed in Section 3.2, the laser beam trajectories ${\vec{x}}_{0}^{k}\left(t\right)$ are discretized as sequences of piecewise linear trajectories $\left[{\vec{x}}_{0}^{k}\left(0\right),{\vec{x}}_{0}^{k}\left(t_{1}\right),\cdots ,{\vec{x}}_{0}^{k}\left(T_{f}\right)\right]$, as described in [31]. The only requirement for this discretization is the fact that all laser beam trajectories must share the same time discretization, i.e., $t_{0},t_{1},\cdots ,T_{f}$ are the same for all trajectories ${\vec{x}}_{0}^{k}$.
2. **Compute the Laplacian eigenvalues:** The Laplacian eigenvalues of the sheet are computed as per Equation (8). Since the eigenvalues $\omega_{ij}$ are time-independent, this step is performed before the simulation loop starts.
3. **Initialize time and sheet temperature:** The simulation time is initialized to $t_{0}\leftarrow 0$. In order to satisfy the initial temperature condition $u\left(0\right)=u_{\infty }$, the pseudo coefficients are initialized as $\theta_{ij}^{k}\left(0\right)=0$ (see Equation (4)).
4. **Update current time *t*:** The current simulation time $t=t_{l+1}$ is updated according to the previous time $t_{0}=t_{l}$, in concordance with the discretization of trajectories from step 1.
5. **For each laser beam *k*:** This inner loop computes the pseudo-coefficients $\theta_{ij}^{k}\left(t\right)$ for each laser beam (k=1⋯num_lasers).
6. **Question: Is laser beam *k* turned on?:** This step allows for simulating asynchronous laser beams by asking at the current time *t* if the laser is turned on/off. Therefore, each laser beam might have its own internal time frame $\left[t_{0}^{k},t_{f}^{k}\right]$, different from the simulation time frame $\left[0,T_{f}\right]$.
7. **Set power $P_{k}$/Set null power $P_{k}\leftarrow 0$:** In the previous step, the program checks the state (on/off) of the current laser beam *k*. The laser is turned on by the simulation by setting its corresponding power input $P_{k}$. In the case of the laser being turned off, the simulation simply sets its power to 0.
8. **Compute the pseudo-coefficients for each laser beam:** The pseudo-coefficients in Equation (7) are solved analytically for each laser beam as described in [31]. The number of coefficients computed is truncated to num_coeffs.
9. **Question: k<num_lasers?:** Check if the pseudo-coefficients have been computed for all the laser beams.
10. **Compute the Fourier coefficients as per Equation (6):** This step computes the real Fourier coefficents $\Theta_{ij}\left(t\right)$ from the pseudo-coefficients $\theta_{ij}^{k}\left(t\right)$ as per Equation (6).
11. **If required, compute temperature:** The temperature field is computed on a set of discrete points sampled on the sheet $\left[\left(x_{0},y_{0}\right),\left(x_{1},y_{1}\right)\cdots \right]$ as per Equation (4). The number of coefficients used to compute the solution is truncated to num_coeffs. This step is optional since the result may be stored in the frequency domain ($\Theta_{ij}\left(t\right)$). Therefore, the temperature is made available only when requested by the user, allowing for skipping iterations of no interest and allowing non-monotonic access to the transient temperature history, improving the performance in the process.
12. **$t<T_{f}$?:** Check if the simulation has reached the final step.
13. **END SIMULATION**

There are several concepts in the previous algorithm that help to improve its efficiency, crucial for the interactive nature of the simulation:

1. In step 1 of the previous algorithm, the curved laser beam trajectories ${\vec{x}}_{0}^{k}\left(t\right)$ are discretized as piecewise linear ones $\left[{\vec{x}}_{0}^{k}\left(0\right),{\vec{x}}_{0}^{k}\left(t_{1}\right),\cdots ,{\vec{x}}_{0}^{k}\left(T_{f}\right)\right]$, which inherently produces the time discretization $\left[0,t_{1},\cdots ,T_{f}\right]$. This time discretization does not affect the numerical accuracy of the temperature solution. Therefore, as opposed to FEA (Finite Element Analysis), the time step size $\Delta t=t_{l+1}-t_{l}$ of our algorithm can be arbitrarily large.
2. Step 6 allows turning on/off any laser beam at any point of the simulation, allowing complete asynchronicity between the multiple laser beams. In addition, the algorithm allows to change any laser parameters at will, resulting in time-dependent parameters $P_{k}\left(t\right)$ (laser power) and $r_{k}\left(t\right)$ (spot radius). For simplicity, this manuscript uses constant parameters.
3. The complete information of the solution is stored in the frequency domain (step 10) and temperature data is computed only when requested. Therefore, the user requests the temperature only at specific times and in specific zones (i.e., at the middle or the end of the simulation). This frequency-based approach has the advantage of providing non-monotonic access to the temperature history, allowing arbitrary simulation time-location requests. Furthermore, since the space discretization does not affect the solution, any sheet sampling can be used to render the temperature (rectangular grid, triangular mesh, a curve or a single point in the sheet).
4. In step 10, each pseudo-coefficient $\theta_{ij}^{k}$ is independent from the rest of the pseudo-coefficients (Equation (6)). Similarly, in step 11, the temperature value *u* at a given point $\vec{x}$ is independent from the temperature on the rest of the sheet (Equation (4)). Therefore, the computation in both of these steps is parallelized.

## 4. Results

This section presents the simulation results of our algorithm. For all the simulations, a Fourier discretization of 512×512 coefficients and a spatial (grid) discretization of 512×512 points are used. These resolutions have been chosen as they have shown in our experiments enough accuracy within our desired execution time ranges (see Section 4.1 and Section 4.3, respectively). As the ratio $\frac{\max(a,b)}{\min_{k}\left(r_{k}\right)}$ (sheet size vs. spot size) increases, it could be necessary to increase the number of Fourier coefficients. However, a sensitivity analysis of our algorithm’s discretization is out of the scope of this manuscript.

Table 1 presents the geometric and physical parameters of the sheet used in the simulations. All of the experiments presented in this manuscript do not consider the effect of heat reflected by the sheet (R=0). The first study case presents two different laser beams heating the sheet simultaneously. The first laser beam follows a star-shaped trajectory on the sheet while the second laser beam follows a rectangular trajectory. Table 2 presents the parameters of each laser beam. As discussed in Section 3, our algorithm not only enables different parameters for multiple lasers (path, power, speed, spot radius), but it also allows independent time frames for each trajectory. Parameter values for Table 1 and Table 2 are taken from state-of-the-art literature [14,31,35]. However, laser beam power has been down-scaled to account for sheet heating instead of cutting or bending.

**Figure 3:**
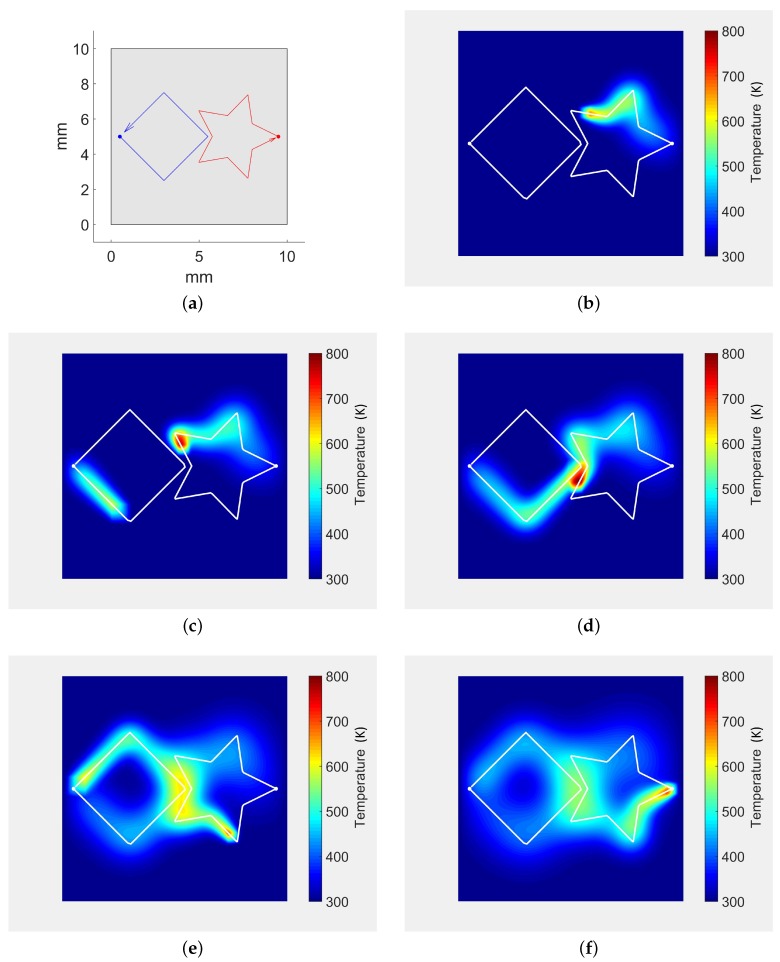
Laser trajectories and sheet temperature history for simultaneous diverse shape laser trajectories. (**a**) laser trajectories for the simulation; (**b**) t=0.060 s. Only Star trajectory occurs; (**c**) t=0.075 s. Square trajectory enters the simulation; (**d**) t=0.094 s. Lasers are simultaneously near each other; (**e**) t=0.13 s. Square trajectory finishes. Star trajectory continues; (**f**) t=0.166 s. Star trajectory finishes.

Figure 3 plots the simulation results for two laser beams heating the sheet surface. Figure 3a shows the star and square laser trajectories planned on the sheet. The laser beam parameters for each trajectory are described in Table 2. At the beginning of the simulation, a unique laser beam (star) heats the surface (Figure 3b). As discussed in Section 3.3, our algorithm allows independent time frames for the multiple laser beams. Therefore, the second laser is introduced in the simulation at t=0.065 s (Figure 3c). Figure 3d plots the temperature when the two lasers are near each other (the trajectories do not intersect). Finally, both laser beam trajectories end at different steps: $t_{f}^{k}=0.13$ s for the square trajectory (Figure 3e) and $t_{f}^{k}=0.16$ s for the star trajectory (Figure 3f).

### 4.1. Comparison with FEA

To validate the analytic approach, FEA simulation is performed. The FEA linear system for Equation (1) becomes [31]:

(9) $$\left[\left(\frac{\rho c_{p}}{\Delta t}+\frac{h}{\Delta z}\right)M+\kappa L\right]U^{\left(t+\Delta t\right)}=M\left(\frac{\rho c_{p}}{\Delta t}U^{\left(t\right)}+\frac{1}{\Delta z}\sum_{k=1}^{num\_lasers}\int_{t}^{t+\Delta t}F_{k}^{\left(\tau \right)}d\tau +\frac{h}{\Delta z}u_{\infty }\right),$$

where U and $F_{k}$ are the vectors of temperature and heat sources sampled on the sheet, and L and M are the stiffness and mass matrices, respectively [31].

The software ANSYS^®^ Academic Research Mechanical, Release 17.2, is used to perform the FEA simulations. ANSYS^®^ element SHELL131 is employed. Element thickness and material properties are set as per Table 1. The elements are configured to have a constant temperature along the thickness and to evaluate convection at the sheet surface, as per Equation (1). To represent the area heated by the laser beams at every time step, ANSYS^®^ surface loads (Heat Fluxes) are applied on the FEA elements that lie inside the heated zone.

**Figure 4:**
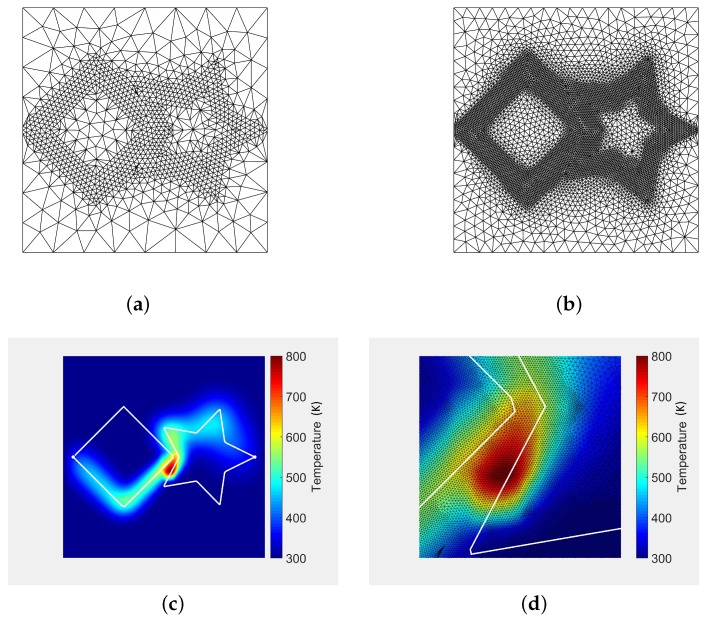
ANSYS^®^ FEA simulation results for the same test case presented in Figure 3. (**a**) coarse FEA mesh (2.1 k triangles); (**b**) intermediate mesh refinement at laser trajectory (13 k triangles); (**c**) *t* = 0.094 s. FEA temperature; (**d**) 10x zoom on Figure 4c.

Figure 4 plots the FEA results for the study case presented in Table 2. The mesh of the domain is generated so that it is more dense in the neighborhoods of the laser trajectories. Figure 4a shows the initial triangular mesh computed using the FEA software which is then refined several times (Figure 4b) to improve the numerical accuracy of the solution. After five re-meshing iterations, the final mesh contains 126 k triangles and 63 k nodes. Figure 4c plots the FEA temperature at the moment the two laser beams get closer to each other (t=0.094 s). The temperature peak is in the middle of the two trajectories (Figure 4d), due to both paths not intersecting each other.

**Figure 5:**
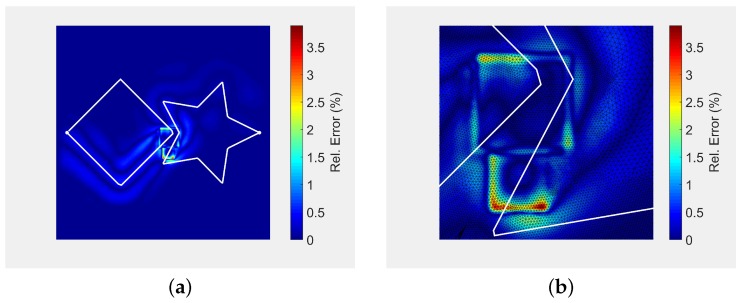
Appraisal of our analytic solution (Figure 3d) vs. the FEA solution (Figure 4c). (**a**) relative error. Analytic solution w.r.t. FEA (max. relative error: 3.9%); (**b**) 10x zoom on Figure 5.

Figure 5 plots the relative error distribution of our analytic solution (Figure 3d) with respect to FEA (Figure 4c). The maximum relative error is 3.9%, located around the laser spots (Figure 5b). This small deviation between our method and FEA arises because: (a) the number of Fourier coefficients used in implementation of Equation (4) affect our algorithm precision, (b) the precision of the FEA algorithm is dependent on mesh and time resolution, element type, etc., and (c) the FEA approach approximates the laser spots using FEA elements at each time step, not preserving the exact geometry of the laser spots. The small square shape of the error in Figure 5b is due to the squared laser model presented in Equation (3).

### 4.2. Multiple Laser Beams

**Figure 6:**
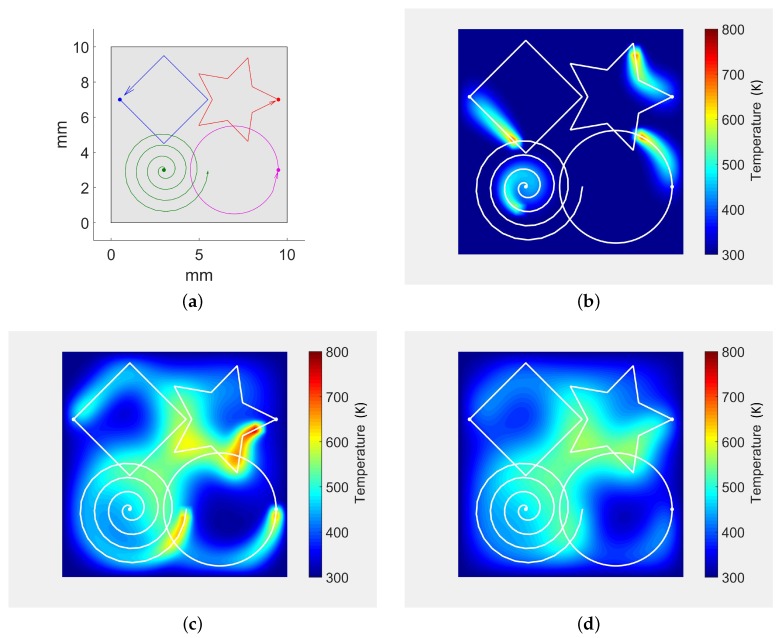
Laser trajectories and temperature history for time and space overlapping trajectories. (**a**) planned laser trajectories: Square, Star, Spiral, Circle; (**b**) t=0.03 s. All trajectories start at t=0 s; (**c**) t=0.158 s. Square and Circle trajectories finished; (**d**) t=0.2 s. All trajectories finished. The sheet cools down.

The algorithm introduced in this manuscript allows multiple laser beams heating the surface at the same time. This section presents additional experiments with more than just two laser beams. Figure 6 presents a study case with four simultaneous laser beams drawing different shapes on the sheet: a square, a star, a spiral and a circle trajectory (Figure 6a). All of the laser beams have the same parameters (power and radius) and start at the same time (Figure 6b). However, they do not finish at the same time (Figure 6c).

As discussed in Section 3.3, our algorithm allows for defining different independent time frames for each laser beam by turning on/off specific laser beams. Such approach even allows to turn off all the laser beams and continue the simulation. Figure 6d illustrates this approach by continuing the simulation after all the laser beams have finished their trajectories, where only thermal conduction and thermal convection are taken into account.

**Figure 7:**
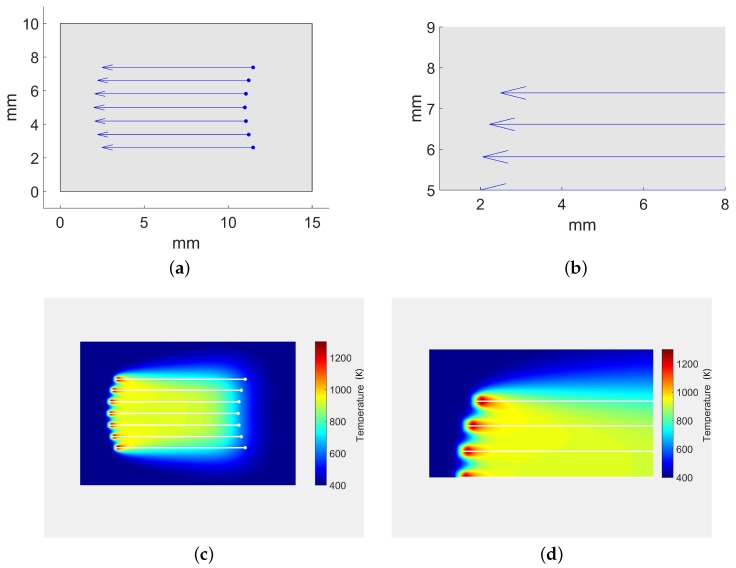
Laser trajectories and temperature results for the simulation case presented in [33]. Similar simulation parameters have been used to replicate the experiment. (**a**) complete sheet. Simultaneous linear trajectories based on [33]; (**b**) laser trajectories (zoom near laser spots); (**c**) temperature map (full sheet); (**d**) temperature map (zoom).

In order to visually compare our algorithm with other simulation approaches in the literature, the study case presented in [33] is replicated in this manuscript (Figure 7). In this study case, seven simultaneous lasers are distributed uniformly on the *y*-axis. Each laser beam follows a straight line trajectory and together they draw an arc in the heating front (Figure 7a,b). Our algorithm is able to produce similar results to [33] despite the simplification of the analytic model (Figure 7c,d).

**Figure 8:**
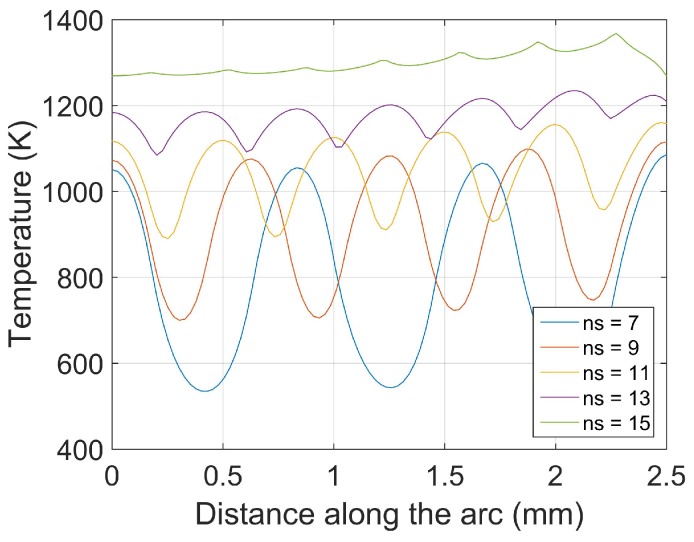
Temperature along the arc for the simulation presented in Figure 7. Our results are similar to those in [33]. ns represents the number of lasers.

Figure 8 plots the temperature distribution along the arc for different number of laser beams. As the number of laser beams increase (and the arc length remains the same), the oscillations of the temperature are mitigated and the temperature increases. Such a result is consistent with the analysis presented in [33]. As discussed in Section 3.3, our algorithm allows for computing easily the temperature along the arc without even requiring to calculate the temperature on the rest of the sheet, which improves the computation time for this particular case.

### 4.3. Performance Assessment

This section evaluates the performance of our algorithm under hardware-accelerated (GPU) and non-accelerated (CPU) platforms. The presented algorithm has been implemented using the OpenCL framework to create an optimized solution that targets both CPU and GPU. On systems where only a CPU is available, our implementation makes use of multi-core parallelization and vectorization to speed-up computation. On systems where a GPU is available, the memory hierarchy can be explicitly controlled using the OpenCL API. The workload is divided into small groups, in order to exploit reuse of computed data using local memory. In this manner, a high speed-up is achieved due to both efficient use of memory and massive parallelization.

**Figure 9:**
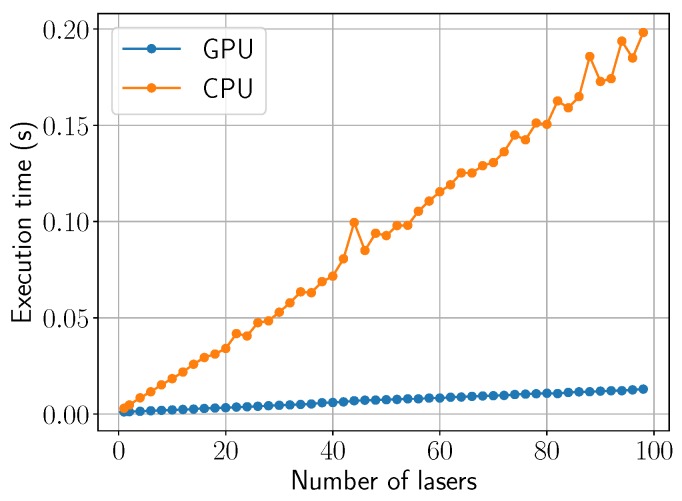
Comparison of CPU and GPU execution times (s) for the computation of 512×512 Fourier coefficients as the number of lasers increase.

The performance results have been measured with the following test platform: A desktop PC using Windows 10 OS with an Intel^®^ Core^TM^ i5-6500 (CPU), 8 GB RAM and NVIDIA^®^ GeForce^®^ GTX 960 graphics card. Our algorithm is able to simulate any number of laser beams. Figure 9 plots the execution times for the computation of the Fourier coefficients as a function of the number of laser beams. The figure compares the execution times of the CPU against the GPU to compute 512×512 Fourier coefficients. The computational cost increases with a large slope in the CPU approach while being nearly constant in the GPU approach. The more laser beams are added, the more it benefits from GPU parallelization. In addition, the computation of the Fourier coefficients in the GPU is preferable. In this way, there is no need to transfer the coefficients back and forth from host-to-device on each iteration since they always stay in GPU memory. For this analysis, a single time step has been considered instead of the whole simulation.

**Figure 10:**
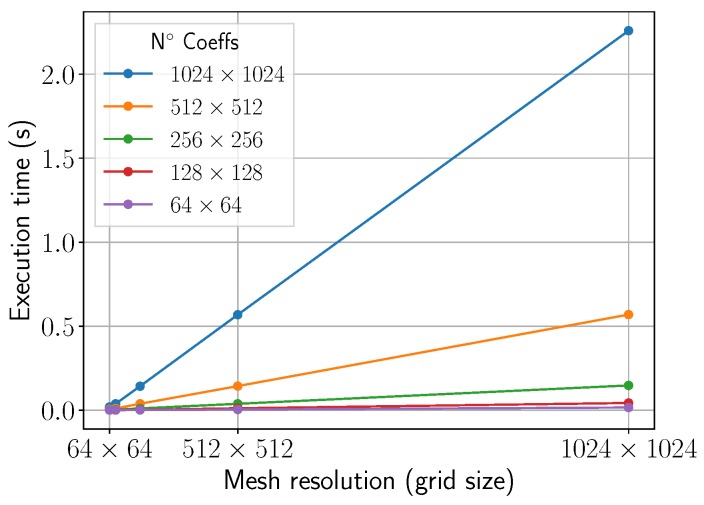
Execution times (s) for the computation of the temperature as the resolution (mesh size) increases. Results for different number of Fourier coefficients are presented.

Figure 10 shows the computation times of the temperature retrieval in a mesh grid as a function of the grid size. While the curves for Fourier resolutions (number of coeffs) below 512×512 display a computational cost relatively low (<0.5 s) for any spatial resolution, Fourier resolutions above that point (1024×1024) become expensive (>0.5 s) for our simulation purposes. Therefore, on our test platform, we have observed that a good balance between accuracy and computation time can be achieved by setting a resolution of 512×512 for both the frequency (Fourier) and spatial (grid) domains. Compared to the previous work [31], a 10x speed-up is achieved at the temperature evaluation by using local GPU memory.

Additionally, since the computation of the temperature is not compulsory at each iteration of the algorithm (as discussed in Section 3.3), the simulation may ignore irrelevant time steps (defined by the user). Moreover, the independence of the algorithm accuracy from the spatial discretization allows the user to specify specific domains (e.g., a sheet section, a curve or a single point) without requiring the whole sheet temperature. These aspects improve the computation efficiency of the simulation for specific requirements of the user.

### 4.4. Integration within an Interactive Laser Cutting Simulation Environment

Nowadays, multi-laser machines with multi-trajectory capabilities do not represent a significant share of the market. The current state-of-the-art laser cutting machines can be divided in three main groups: (1) multi-laser machines with independent and disjoint working areas for each laser head, (2) multi-laser machines with parallel heads working simultaneously and (3) multi-laser machines with fully individual and autonomous laser heads (whose trajectories may intersect or get close enough). The first scenario can be reduced to a collection of mono-laser machining scenarios, since the individual working areas for each laser head avoid interference with the neighbouring laser heads and their heat effects. Therefore, the approach introduced in [31] is still valid to simulate the temperature for each laser head of these multi-laser machines.

In the second type of multi-laser machines, all laser heads receive the same trajectories and machining commands, but their separation or offset can be setup and reconfigured during the machining. Additionally, each laser head can be switched off and on individually. The offset between the heads does not guarantee disjoint working zones. Therefore, the simulation approach presented in this work is used to address the potential interference between the multiple heat sources.

The last type of multi-laser machines use mirrors and lenses to move the spot of the available laser heads. Each laser spot can be commanded independently of the others, even allowing two or more spots to converge at the same physical point on the sheet metal. The methodology presented in this work is used to simulate such scenarios.

In the context of the second scenario, this section presents an interactive simulator of a laser cutting machine with three laser heads. All laser heads are arranged side by side in the bridge of the machine (*x*-axis of the machine). Each individual laser head can be switched on and off individually and their relative positions among them (*y*-axis of the machine) can be changed by means of specific machining instructions.

The virtual simulator itself uses a contour-based representation to model the geometry of the processed sheet [36,37], presenting a virtual 3D interactive scenario with the multi-laser CNC (Computer Numerical Control) machine that receives the machining instructions. In this virtual scene, the moving and cutting instructions move the corresponding axis of the machines (bridge, laser heads offset along the bridge and laser head height over the sheet metal). The heat sources are then calculated and sent to the heat simulation procedure, updating the temperature of the sheet, which is rendered as a texture over the virtual sheet metal.

Since all the laser heads receive the same machining instructions (although their position along the bridge differ), all movements start and end at the same time. As discussed in Section 3.3, the laser beam trajectories must share the same time discretization, which in this case, is guaranteed by design. If a unrestricted multi-laser machine is used with independent laser heads, i.e., receiving different machining instructions, a global time clock must be used in order to trigger the update of the temperature computation with the correct positions of the moving laser heads.

The introduction of the multi-laser machines in the industrial world has an impact from the design point of view. Even with just one laser, the designer of the NC programs must take into account the expected produced heat and how it spreads over the sheet metal in order to optimize the nesting of the produced parts. With a multi-laser machine, this procedure is even more critical, as the resulting heat of the multiple sources can accumulate in some areas. NC designer is expected to use the presented virtual simulator to visualize how the cutting process produces the parts and to analyze the computed temperature through the sheet. If any situation becomes problematic, the designer would make changes to the NC program in order to address the situation.

The presented multi-laser simulator runs at interactive rates. Therefore, the designer can improve the optimization workflow, as many simulations can be run in a short period of time. In contrast, high quality simulations with FEA software, although being highly precise, are computationally expensive. Thus, the number of test configurations that the designer can test during the design phase is limited. Nevertheless, at the end of the optimization phase, the interactive heat simulations can be complemented with high quality FEA simulations.

From the industrial point of view, this improved design workflow, assisted with the interactive multi-laser simulation, (1) provides better NC programs in terms of the quality of the produced parts, (2) produces economical benefits due to shorter machining times or less wasted resources, and (3) improves the safety of the operators in the factory floor, reducing unnecessary risks due to unexpected behaviour of the NC programs.

**Figure 11:**
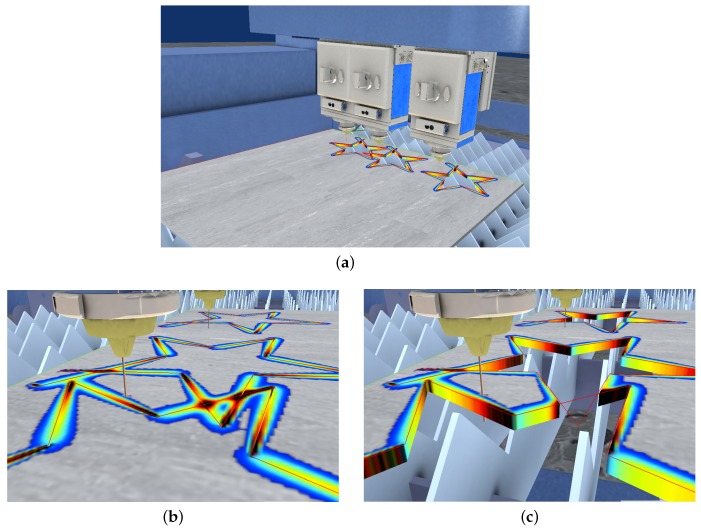
Multi-laser machining in the interactive simulator. A star shape is machined by the three laser heads. (**a**) virtual multi-laser machine; (**b**) temperature shown on the sheet surface; (**c**) geometric cut of the sheet.

Figure 11a shows a machine with three laser heads working simultaneously, i.e., they receive the same machining instructions with fixed spatial offsets). Each laser head machines a star figure. The stars machined by the first and second laser heads overlap, while the third laser head produce an isolated star figure. Figure 11b shows a closer view of the cutting area while Figure 11c shows the same viewpoint, but the cut stars removed from the visualization. The simulation results show that intersecting trajectories present temperature peaks (black zones) where the trajectories overlap (see Figure 11b).

## 5. Conclusions

This manuscript presents a novel methodology for the simulation of heat transfer on rectangular sheet metal under multi-laser beam heating. The algorithm is based on an analytic frequency-based solution to the heat transfer equation which considers some simplifications (2D domain, constant material parameters) in favor of simulation speed. Such simplifications allow the algorithm to solve the transient temperature map on large space/time domains and complex laser trajectories. Furthermore, the algorithm allows many simultaneous laser beams with independent parameters (laser power, speed and spot radius) and time frames (i.e., each laser beam can be turned on/off during the simulation). The numerical accuracy of the algorithm is independent from the space/time discretization, allowing non-monotonic access to the temperature time history and arbitrary discretization of the sheet domain. Our simulation tests show that the algorithm is able to render correctly the temperature maps for several laser beams with different trajectories using mesh grids. A numerical comparison with FEA shows that our algorithm solution deviates from the FEA one a maximum of 3.9% and only around the laser spot. An assessment of the algorithm performance shows that, in an implementation using GPUs, the number of laser beams barely affects the simulation time. In addition, compared to previous work [31], the temperature retrieval time has been reduced by a factor of ten. Finally, the algorithm is implemented into an interactive laser cutting simulation environment for the assessment in real time of laser cutting processes in real manufacturing scenarios.

The presented algorithm simplifies by design the mathematical model of the problem in favor of interactive simulations. As future work, we work on: (1) simulating the nonlinear behaviour of the material properties which arises due to the high temperature gradients, (2) simulating physically the laser ablation and material removal in sheet cutting processes, (3) coupling the model with an analytic stress model in order to evaluate the potential structural damage due to thermal stress, and (4) studying the sensitivity of the Fourier discretization with respect to parameters such as sheet size and radii of the laser spots.

## Figures and Tables

**Table 1:** Sheet heat transfer parameters for the simulations (same as in [14,31,35]).

Parameter	Description	Value
**Geometry**		
*a*	width	0.01 m
*b*	height	0.01 m
Δz	thickness	0.001 m
**Material**	**AISI 304 Steel**	
ρ	density	8030 $\mathrm{kg}/m^{3}$
$c_{p}$	specific heat	574J/(kg·K)
κ	thermal conductivity	20W/(m·K)
*R*	reflectivity	0
**Convection Type**	**Natural**	
*h*	convection coefficient	$20\;W/(m^{2}\cdot K)$
$u_{\infty }$	ambient temperature	300K

**Table 2:** Parameters for the simultaneous laser heating trajectories of Figure 3a (similar to [14,31,35]).

Parameter	Description	Star Trajectory	Square Trajectory
$t_{0}^{k}$	start time of trajectory	0.00 s	0.06 s
$t_{f}^{k}$	end time of trajectory	0.16 s	0.13 s
$P_{k}$	power	100 W	200 W
$r_{k}$	spot radius	0.0003 m	0.0005 m
$∥{\vec{v}}_{k}∥$	trajectory speed	0.1 m/s	0.2 m/s

## Abbreviations

The following abbreviations are used in this manuscript:

FEA/FEM	Finite Element Analysis/Finite Element Method.
GPU	Graphics Processing Unit.
a,b,Δz	Width, height and thickness of the sheet (*m*).
$\vec{x},t$	Spatial $\vec{x}=\left(x,y\right)\in \left[0,a\right]\times \left[0,b\right]$ and temporal $0\leq t\leq T_{f}$ (s) coordinates for the simulation.
$u=u(\vec{x},t)$	Temperature distribution along the sheet at any given time (K).
ρ	Sheet density (kg/m${}^{3}$).
$c_{p}$	Sheet specific heat (J/(kg K)).
κ	Sheet thermal conductivity (W/(m K)).
*R*	Sheet reflectivity (0≤R<1).
q=q(u)	Temperature-dependent heat convection on the sheet surface (W/m${}^{2}$).
$u_{\infty }$	Ambient temperature *K*.
*h*	Natural convection coefficient (W/(m${}^{2}$ K)).
$f_{k}$	Heat input from laser beam *k* (W/m${}^{2}$). k=1⋯num_lasers.
$P_{k}$	Power of laser beam *k* (W).
$r_{k}$	Radius of laser spot *k* (m).
${\vec{x}}_{0}^{k}={\vec{x}}_{0}^{k}\left(t\right)$	Laser spot center for laser beam *k* at time *t*.
$\left[t_{0}^{k},t_{f}^{k}\right]$	Simulation time frame in which the laser beam *k* remains turned on. $0\leq t_{0}^{k}<t_{f}^{k}\leq T_{f}$.
${\vec{v}}_{k}$	Scan speed of laser beam *k* (m/s).
$F=F(\vec{x},t)$	Sum of all laser beam heat sources (W/m${}^{2}$).
$X_{i}=X_{i}\left(x\right)$	Fourier basis function associated to the *x* coordinate. i=1⋯∞.
$Y_{j}=Y_{j}\left(y\right)$	Fourier basis function associated to the *y* coordinate. j=1⋯∞.
$\Theta_{ij}=\Theta_{ij}\left(t\right)$	Fourier coefficient associated to basis functions $X_{i}$ and $Y_{j}$.
$\theta_{ij}^{k}=\theta_{ij}^{k}\left(t\right)$	Pseudo-Fourier-coefficient associated to the k-th laser beam source.
$\omega_{ij}$	ij-th eigenvalue of the heat operator (Laplacian) on the rectangular sheet.
